# An Organic Solvent-Tolerant Lipase with Both Hydrolytic and Synthetic Activities from the Oleaginous Fungus *Mortierella echinosphaera*

**DOI:** 10.3390/ijms19041129

**Published:** 2018-04-10

**Authors:** Alexandra Kotogán, Carolina Zambrano, Anita Kecskeméti, Mónika Varga, András Szekeres, Tamás Papp, Csaba Vágvölgyi, Miklós Takó

**Affiliations:** 1 Department of Microbiology, Faculty of Science and Informatics, University of Szeged, Közép fasor 52, H-6726 Szeged, Hungary; primula15@gmail.com (A.Ko.); czambranocarrillo@gmail.com (C.Z.); kecskemeti.anita@gmail.com (A.Ke.); varga.j.monika@gmail.com (M.V.); szandras@bio.u-szeged.hu (A.S.); pappt@bio.u-szeged.hu (T.P.); csaba@bio.u-szeged.hu (C.V.); 2 MTA-SZTE “Lendület” Fungal Pathogenicity Mechanisms Research Group, University of Szeged, Közép fasor 52, H-6726 Szeged, Hungary

**Keywords:** extracellular lipase, zygomycetes, biochemical characterization, *p*-nitrophenyl esters, alkyl esters, esterification, transesterification, immobilization

## Abstract

Lipase enzymes of the oleaginous fungal group *Mortierella* are rarely studied. However, considering that most commercial lipases are derived from filamentous fungal sources, their investigation can contribute to the cost-effective development of new biotechnological processes. Here, an extracellular lipase with a molecular mass of 30 kDa was isolated from *Mortierella echinosphaera* CBS 575.75 and characterized. The purified lipase exhibited an optimal *p*-nitrophenyl palmitate (*p*NPP)-hydrolyzing activity at 25 °C and pH 6.6–7.0 and proved to be highly stable at temperatures up to 40 °C and under broad pH conditions. The enzyme was active under low temperatures, retaining 32.5% of its activity at 10 °C, and was significantly stable in polar and non-polar organic solvents. The *K*_m_, *V*_max_, and *k*_cat_ for *p*NPP were 0.336 mM, 30.4 μM/min, and 45.7 1/min for *p*NPP and 0.333 mM, 36.9 μM/min, and 55.6 1/min for *p*NP-decanoate, respectively. The *p*NPP hydrolysis was inhibited by Hg^2+^, *N*-bromosuccinimide, and sodium dodecyl sulfate, while ethylenediaminetetraacetic acid and metal ions, such as Ca^2+^, Mg^2+^, Na^+^, and K^+^ enhanced the activity. The purified lipase had non-regioselective activity and wide substrate specificity, showing a clear preference for medium-chained *p*-nitrophenyl esters. Besides its good transesterification activity, the enzyme appeared as a suitable biocatalyst to operate selective esterification reactions to long-chained alkyl esters. Adsorption to Accurel MP1000 improved the storage stability of the enzyme at 5 °C. The immobilized lipase displayed tolerance to a non-aqueous environment and was reusable for up to five cycles without significant loss in its synthetic and hydrolytic activities. These findings confirm the applicability of both the free and the immobilized enzyme preparations in future research.

## 1. Introduction

Lipases (E.C. 3.1.1.3) catalyze the hydrolysis and the synthesis of ester bonds and are an important group on the global enzyme market. They are effective biocatalysts used in many sectors, such as in the food, pharmaceutical, organic synthesis, and detergent industries [1]. The direction of the lipase reactions is influenced by the water activity of the reaction media; thus, esterification, trans- and interesterification occur under low water content or non-aqueous conditions. The main advantages of using lipases for ester synthesis are that they require milder reaction conditions and result in purer products than the alternative chemical procedures. Thus, downstream treatment is generally not necessary, which makes the production more economical and eco-friendly [2]. Additionally, the application of lipases, which retain their activity and stability in organic solvents, could enhance substrate solubility and product recovery during enzymatic synthesis reactions [3].

Most current commercial lipases are derived from microbial sources produced by bacteria or filamentous fungi [4]. However, many lipases often display low activity or specificity towards special or non-natural substrates and can show low stability under operational conditions in industrial processes [1]. Therefore, identification and characterization of novel microbial lipases with promising hydrolytic and/or synthetic properties have special importance for industrial and biotechnological process development purposes.

Mucoromycota (former Zygomycota) fungi are good sources of lipases and can be useful for sustainable enzyme and biofuel production or hydrocarbon biodegradation [5]. In this group of filamentous fungi, members of the genus *Mortierella* are extensively studied due to their ability to accumulate lipid bodies containing triacylglycerols and fatty acids. Several oleaginous species are utilized industrially to produce polyunsaturated fatty acids with different carbon chain lengths and saturations [6]. Lipases may play a role in lipid accumulation, the formation of lipid bodies, and the improvement of their composition [7,8,9]. Biochemical characterization of these enzymes may contribute to the knowledge of the lipid metabolism of these fungi and may provide data on new lipases with potential biotechnological interest. However, *Mortierella* lipases have been given less attention and very few data are available about their synthetic properties [10]. To our knowledge, only one intracellular and one extracellular lipase have been isolated from this genus so far [11,12].

In our previous works, the extracellular lipase production of several Mucoromycota strains were tested, and the *Mortierella echinosphaera* CBS 575.75 isolate resulted in high enzyme yield in wheat bran-based submerged and solid-state fermentation systems [13]. An investigation of the crude *M. echinosphaera* lipase extract showed promising transesterification and esterification activities in water-free *n*-heptane-based organic conditions [10]. Here, we aimed to isolate and characterize this extracellular lipase, paying attention to its hydrolytic and synthetic activities including the investigation of the esterification catalysis to form different fatty acid methyl esters. Alkyl esters, especially methyl and ethyl esters, are widely used in industry; besides the production of biodiesel and other specific lipids, they are applicable in flavor and aroma ester synthesis as well [14,15]. Because the entrapment of lipases allows for their reuse and can improve their activity and thermal and solvent stability [16], the immobilization of the purified *M. echinosphaera* lipase has also been carried out on a polypropylene hydrophobic support.

## 2. Results and Discussion

### 2.1. Purification of Extracellular Lipolytic Enzyme

**Figure 1:**
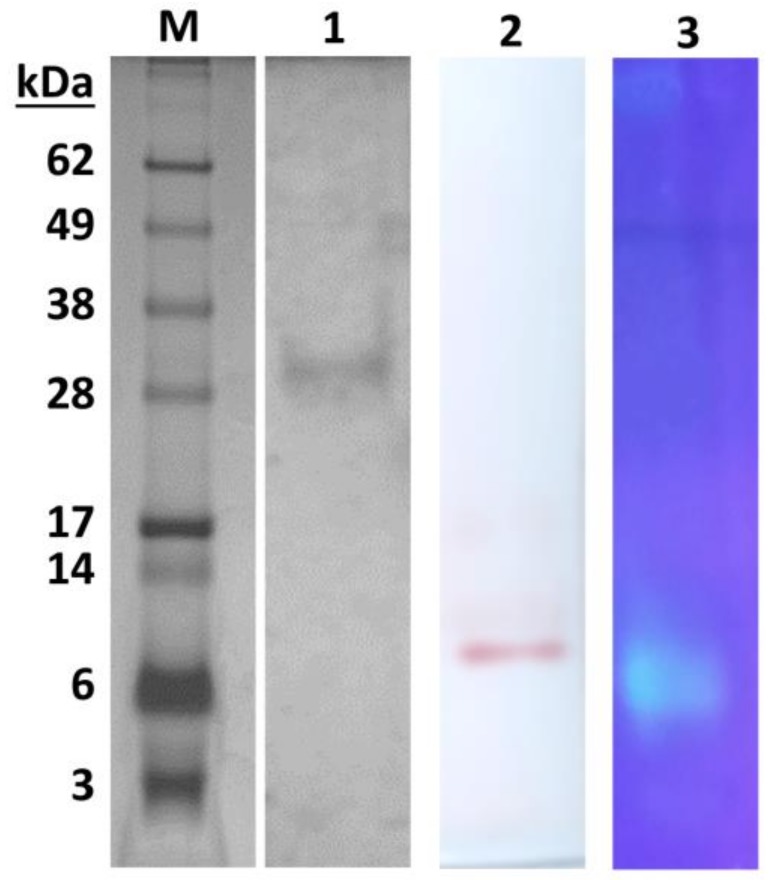
SDS-PAGE and zymogram analysis of the purified lipase from *M. echinosphaera* CBS 575.75. Lane M: See Blue Plus2 SDS-PAGE molecular weight standard (Life Technologies, Carlsbad, CA, USA); Lane 1: purified lipase on a 4–12% SDS-PAGE gel; Lane 2: zymography using α-naphtyl acetate as a substrate; Lane 3: zymography using 4-methylumbelliferyl nonanoate as a substrate.

Although some applications, i.e., in textile, detergent, and biodiesel production, do not require a homogeneous enzyme preparation [17], the purification of lipases to homogeneity is essential for many industrial uses, such as the fine chemical, pharmaceutical, and cosmetic industries [18]. The major limitation of the industrial use of lipases is the high cost of production. Because of this, hemp, flax, poppy, and pumpkin seed residues and oat and wheat bran were tested previously [13,19] as cheap substrates to investigate the lipase production of *M. echinosphaera*. The effect of mineral salts, olive oil, and cultivation time on the product yield was also evaluated in both submerged and solid-state fermentation conditions. Maximum specific activity was detected after four-day fermentation in a wheat bran-based submerged system [13]; therefore, this cultivating condition was applied to produce the extracellular *M. echinosphaera* lipase in high yield. From the ferment broth, the enzyme was purified by ammonium sulfate precipitation, and gel filtration and anion exchange chromatographies resulted in 19.5-fold purification with a recovery rate of 0.17% (Table 1). The relatively low final recovery can be ascribed to the insoluble lipase aggregates that may have formed during the ammonium sulfate fractionation and/or the olive oil inductor added to the submerged fermentation medium [12,13]. The purified lipase exhibited a specific activity of 378.57 U/mg during the hydrolysis of *p*-nitrophenyl palmitate (*p*NPP). The molecular weight of the enzyme was estimated to be about 30 kDa by sodium dodecyl sulfate polyacrylamide gel electrophoresis (SDS-PAGE) (Figure 1). Zymogram analysis of the purified enzyme showed a single active lipase band stained with α-naphthyl acetate and 4-methylumbelliferyl nonanoate (Figure 1). The purified *M. echinosphaera* lipase has a neutral isoelectric point of 6.2. The transesterification activity of the purified enzyme between *p*NPP and ethanol proved to be 18.6 U/mg in *n*-heptane at 40 °C.

### 2.2. Characterization of the Purified Lipase

#### 2.2.1. Effect of the Temperature and pH on the Activity and Stability of the Purified Lipase

**Figure 2:**
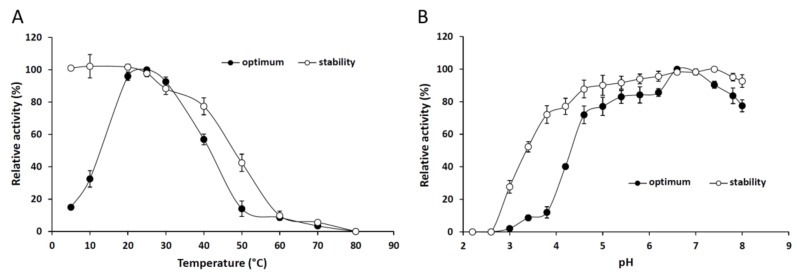
Effect of the temperature (**A**) and pH (**B**) on the activity and stability of the purified *M. echinosphaera* CBS 575.75 lipase. To determine the optimum temperature, *p*NPP-hydrolyzing activity was measured in a temperature range of 5–80 °C in phosphate buffer (50 mM, pH 6.8) for 30 min. Thermostability studies were done by measuring the residual activity after preincubation of the enzyme at different temperatures for 4 h in phosphate buffer (50 mM, pH 6.8). The effect of pH on *p*NPP-hydrolyzing activity was determined at 25 °C using 50 mM McIlvaine buffer with pHs ranging from 2.2 to 8.0. For pH stability, the residual activity was evaluated after preincubation of the enzyme at the various pHs for 24 h at 4 °C. Values are means (*n* = 3); error bars indicate standard deviations.

Figure 2A shows that 25 °C proved to be the optimal temperature for maximum lipolytic activity of the purified lipase. In general, the Mucoromycota extracellular lipases isolated so far had an optimum temperature over 25 °C [20,21,22,23]. The enzyme also proceeded well at 30 and 40 °C, exhibiting 92% and 56% relative activities, respectively. A significant decrease in the *p*NPP hydrolysis was measured above 50 °C and less than 20% of the activity could be detected at the temperature range from 50 to 80 °C. However, the enzyme retained 96% and 32% of its activity at temperatures of 20 and 10 °C, respectively. Lipases, which are active at low temperatures, are applicable in various industrial fields including pharmaceutical developments, detergent production and food processing technologies [24]. The enzyme was highly stable at temperatures up to 40 °C, but lost most of its activity at temperatures above 50 °C after a 4-h preincubation (Figure 2A).

Under optimal temperature conditions for 30 min, the optimum pH for the lipolytic action of *M. echinosphaera* lipase was 6.6–7.0 (Figure 2B). The enzyme was active in a wide pH range, showing a relative activity above 50% between pH 4.6 and 8.0. The purified enzyme exhibited broad pH stability retaining more than 50% of its initial activity between pH 3.4 and 8.0 after a 24-h preincubation (Figure 2B). Similarly, wide pH tolerance has been found for the *Mortierella alliacea* intracellular lipase [12]. Since the *M. echinosphaera* lipase was highly stable and active at 25 °C and pH 6.8, further analyses of the enzyme were performed under these conditions.

#### 2.2.2. Determination of Kinetic Constants

The Michaelis-Menten constant (*K*_m_), maximum velocity (*V*_max_) and catalytic rate constant (*k*_cat_) of the purified *M. echinosphaera* lipase were determined for *p*NPP and *p*NP-decanoate substrates under optimal reaction conditions. The hyperbolic plot displayed Michaelis-Menten behavior of the enzyme, and the *K*_m_ and *V*_max_ for *p*NPP were found to be 0.336 mM and 30.4 μM/min while these values were 0.333 mM and 36.9 μM/min for the *p*NP-decanoate, respectively, suggesting a high substrate affinity and catalytic efficiency of the enzyme. The calculated *k*_cat_ values were 45.7 and 55.6 1/min for the *p*NPP and *p*NP-decanoate, respectively. Using *p*NPP as a substrate, similar kinetic parameters has also been documented for the recombinant *Rhizopus chinensis* lipase [25]. Additionally, the *M. echinosphaera* lipase showed about three times lower *K*_m_ constants than those found by our research group on *p*NPP for the *Rhizomucor miehei* and *Rhizopus oryzae* lipases [23], which indicates more affinity to the substrate for the *Mortierella* enzyme.

#### 2.2.3. Determination of the Substrate Specificity

**Figure 3:**
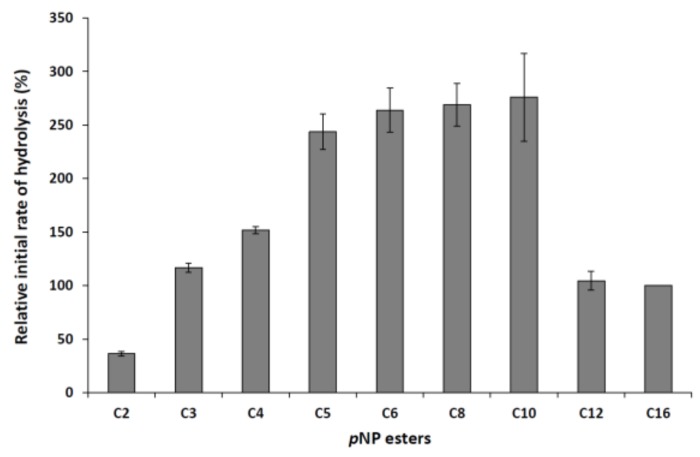
Specificity of the *M. echinosphaera* CBS 575.75 lipase towards *p*NP ester substrates (C2: *p*NP-acetate, C3: *p*NP-propionate, C4: *p*NP-butyrate, C5: *p*NP-valerate, C6: *p*NP-caproate, C8: *p*NP-caprylate, C10: *p*NP-decanoate, C12: *p*NP-dodecanoate, C16: *p*NPP). Reactions were performed at 25 °C for 30 min in 50 mM sodium phosphate buffer (pH 6.8) contained 0.75 mM from the corresponding *p*NP ester substrate. Activity measured with *p*NPP was taken as 100%. Values are means (*n* = 3); error bars indicate standard deviations.

Hydrolysis of aryl esters with different acyl chain lengths (C2–C16) was tested to evaluate the acid specificity of the purified lipase. The *M. echinosphaera* lipase effectively hydrolyzed the substrates containing fatty acids from C3 to C16, indicating a broad substrate specificity for the enzyme (Figure 3). Low activity was observed with the short-chain *p*-nitrophenyl (*p*NP) acetate (C2), resulting in 36% relative initial activity. However, the initial hydrolysis of aryl esters with C5 to C10 fatty acids was about 2.5-fold higher than those detected for *p*NPP, which chromogenic substrate is generally used for lipolytic activity tests. Similarly, specificity for medium-chain fatty acids were observed for other purified Mucoromycota lipases as well, including the enzymes from *Rhizopus homothallicus* [21], *R. miehei* [23], *Rh. chinensis* [26], and *Mucor javanicus* [27].

#### 2.2.4. Positional Specificity

**Figure 4:**
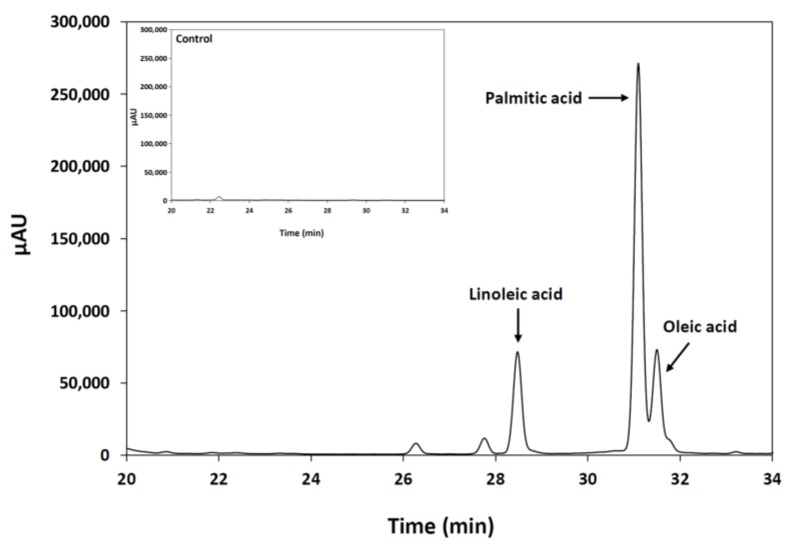
HPLC analysis of the fatty acids formed after the hydrolysis of 1-palmitoyl-2-oleoyl-3-linoleoyl-*rac*-glycerol by the lipase of *M. echinosphaera* CBS 575.75. The enzymatic hydrolysis conditions were pH 6.8, 25 °C and 2-h incubation time. (Inset: control sample contained only 1-palmitoyl-2-oleoyl-3-linoleoyl-*rac*-glycerol).

An important feature of the lipase enzymes is their positional specificity (regioselectivity) observed during the hydrolysis of triacylglycerol molecules. This practical property determines the nature of the di- and monoacylglycerols released during the reaction. In the present study, regioselectivity of the purified *M. echinosphaera* lipase was analyzed through HPLC analysis of the fatty acids released from the hydrolysis of 1-palmitoyl-2-oleoyl-3-linoleoyl-*rac*-glycerol. After incubation at 25 °C for 2 h, linoleic, palmitic and oleic acids were detected with the retention times 28.5, 31.1 and 31.5 min, respectively (Figure 4). Accordingly, the *M. echinosphaera* lipase could hydrolyze all ester bonds (*sn*-1, 2 and 3) of the substrate and belongs to the group of non-regiospecific (non-regioselective) lipases that possess no strict preference for the position. The 1,3- and 2-regiospecific lipases, in contrary, can catalyze the hydrolysis of ester bonds at the *sn*-1,3 and the *sn*-2 positions, respectively. The majority of the lipases are 1,3-regiospecific or non-regiospecific and many fungal lipases, e.g., those of *Fusarium* sp. YM-30, *Geotrichum candidum*, *Penicillium roqueforti* and *Candida curvata*, fall into the non-regiospecific group [28]. Nevertheless, non-regiospecific lipases can be utilized in several biotechnological processes including the catalysis of various transesterification reactions in biodiesel production and the mobilization of saturated fatty acids from plant oils [29,30].

#### 2.2.5. Effect of Metal Ions and Reagents

The effect of various cations (5 mM) and reagents (10 mM) on the purified *M. echinosphaera* lipase activity was also investigated. The *p*NPP hydrolysis was found to be strongly inhibited by Hg^2+^, *N*-bromosuccinimide (NBS) and sodium dodecyl sulfate (SDS) (Table 2). The cation Cu^2+^ had no significant effect on enzyme activity but Co^2+^, Mn^2+^, and Zn^2+^ treatment reduced the *p*NPP substrate hydrolysis by 19–33%. However, the activity increased by about 23, 75, 37, 30 and 42% in the presence of 5 mM Ca^2+^, Mg^2+^, Na^+^, K^+^ and 10 mM ethylenediaminetetraacetic acid (EDTA), respectively. The cations Ca^2+^ and Mg^2+^ in 0.2 mM concentration stimulated the activity of the *Mortierella vinacea* extracellular lipase [11] and the hydrolysis by the intracellular lipase of *M. alliacea* could be improved by using CaCl_2_ in 1 mM concentration [12]. Similarly, Ca^2+^ enhanced the lipolytic activity of *Mucor* sp. [20], *Rhizopus delemar* [31], and *Mucor hiemalis* f. *hiemalis* [32] lipases.

Metal ions can bind to the side chains of amino acids situated on the protein surface, thus stabilizing or destabilizing the enzyme structure and resulting in activity changes [33,34]. For many lipases, it was found that the presence of certain metal ions, particularly divalent cations, is necessary to maintain and enhance their activity and structural stability [35]. Kim et al. [36] monitored the refolding of the recombinant *Pseudomonas fluorescens* lipase, wherein secondary structural changes were induced by Ca^2+^ ions binding to the Asp side chains. Furthermore, increased activity and stability of the protein in trifluoroethanol and dimethyl sulfoxide was also found in the presence of Ca^2+^. In our study, the *M. echinosphaera* lipase has proven to be extremely stable in the presence of several metal salts tested. Moreover, enhanced lipolytic activity was observed in the case of some cations, which can be a promising feature for industrial and biotechnological uses. EDTA did not cause enzyme activity loss, suggesting that specific metal ions are not required for activity and stability.

#### 2.2.6. Effect of Organic Solvents on the Purified Lipase

**Figure 5:**
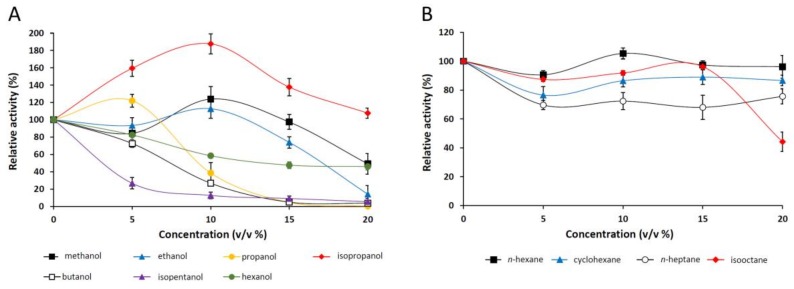
Stability of *M. echinosphaera* CBS 575.75 lipase in the presence of various alcohols (**A**) and alkanes (**B**). The *p*NPP-hydrolyzing activity was determined after 30-min preincubation of the purified enzyme at 25 °C in phosphate buffer (pH 6.8) containing the corresponding solvent (5–20%, *v*/*v*). The activity measured without any solvents was taken to be 100%. Values are means (*n* = 3); error bars indicate standard deviations.

The effect of various alcohols and alkanes on *p*NPP-hydrolyzing activity of the *M. echinosphaera* lipase was studied in the concentration range from 5% to 20% (*v*/*v*) (Figure 5A,B). The enzyme was highly stable in 20% (*v*/*v*) isopropanol and 15% (*v*/*v*) methanol and ethanol presenting 107%, 97%, and 73% relative activities, respectively (Figure 5A). Isopentanol, butanol, and hexanol decreased the enzyme activity in a dose-dependent manner, and about half of the initial activity remained in the presence of 20% (*v*/*v*) hexanol. However, since these alcohols are slightly miscible with water, the preferred dissolution of the *p*NPP substrate in the organic phase may have contributed to this observation. In general, water-miscible solvents have a destabilizing effect on the lipases, causing a decrease in their activity [37]. In our investigations, however, the enzyme activity was enhanced by adding 5% (*v*/*v*) propanol, 10% (*v*/*v*) methanol and ethanol, and up to 20% (*v*/*v*) isopropanol to the standard reaction mixture (Figure 5A). This can be attributed to the better solubility of the substrate, and/or the transesterification reaction initiation supported by a water activity decrease in the surrounding [38,39]. In this context, although we have not investigated the formation of alkyl esters, the released *p*-nitrophenol may be derived from both transesterification and hydrolytic reactions catalyzed simultaneously in the solvent-supplemented medium.

Among alkanes (logP_ow_ > 3.0), *n*-hexane and isooctane had no considerable effect on the *p*NPP hydrolysis of the *M. echinosphaera* lipase up to 15% (*v*/*v*) concentration (Figure 5B). However, 20% (*v*/*v*) isooctane reduced the enzyme activity to 55%. A moderate inhibition with 32% and 23% maximal activity decrease was detected in the presence of *n*-heptane and cyclohexane. This stability in water immiscible solvents was also observed for the *M. echinosphaera* crude enzyme during transesterification reactions testing in our previous study [10]. A cause of this phenomenon could be that the water-immiscible solvents form a hydrophobic surface, which can initiate interfacial activation, thereby stabilizing the active conformation (open form of the lipase) [27]. Furthermore, because the water-immiscible solvents could retain a thin layer of water tightly bound to the enzyme, the hydrophilic surface and thus the native conformation could also be protected [37].

### 2.3. Analysis of Esterification Reactions Catalyzed by Purified Lipase

**Figure 6:**
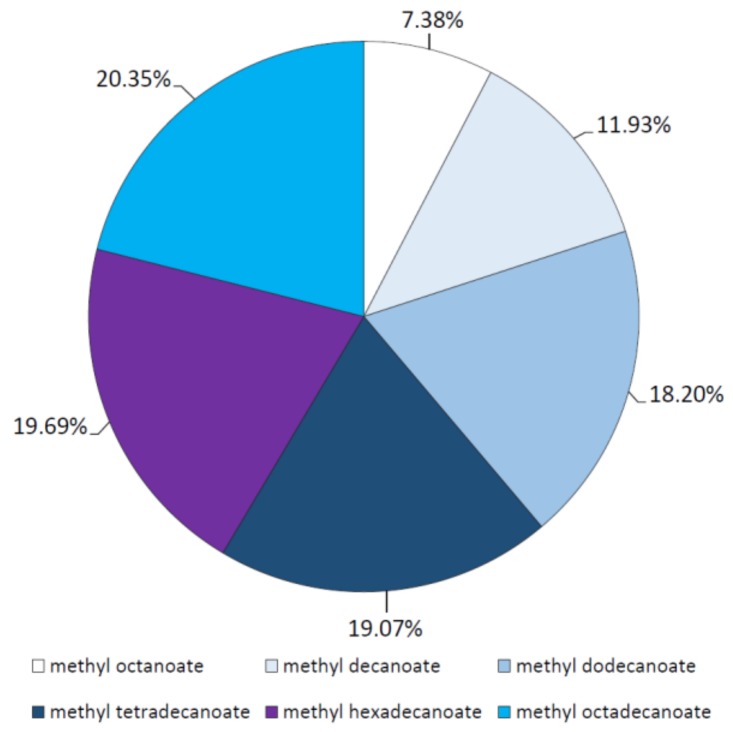
Percent distribution of methyl ester products formed during esterification reactions with the *M. echinosphaera* CBS 575.75 lipase. Reactions were performed at 40 °C for 72 h in *n*-heptane containing 1 M methanol and various fatty acids, each in 10 mM concentration. The total methyl ester yield was taken as 100%.

In these assays, ester synthesis between selected saturated fatty acids from butyric acid (C4) to octadecanoic acid (C18) and methanol was tested in *n*-heptane using the purified *M. echinosphaera* lipase as the catalyst. The reaction mixture was incubated at 40 °C and contained the all individual fatty acid components at the same time. Since the esterification catalyzed by the *M. echinosphaera* crude lipase is significantly slower than the transesterification [10], a 72-h incubation time was used for the reaction. Gas chromatography (GC) analysis of the obtained synthetic esters revealed a diverse affinity of the enzyme to esterify the different fatty acids. Methyl ester formation was not observed with short-chain butyric and hexanoic acid (C4 and C6), but if the length of the fatty acid carbon chain was increased, the ratio of the corresponding methyl ester in the total ester yield also increased. The order of the percent distributions of methyl esters produced were found to be methyl octanoate < methyl decanoate < methyl dodecanoate < methyl tetradecanoate < methyl hexadecanoate < methyl octadecanoate (Figure 6). Accordingly, the purified *M. echinosphaera* lipase has the affinity for esterification of long-chain fatty acids from C12 to C18, especially for the C16 and C18 fatty acids. Silva and Jesus [40] tested the esterification capacity of an immobilized *Mu. javanicus* lipase, in which the highest ester yield was formed in case of the longest fatty acid (dodecanoic acid) involved. Similar to *M. echinosphaera* lipase, the *Rh. chinensis* lipase rather preferred the medium- and long-chain fatty acids (i.e., from C8 to C14) than the short-chain ones in the esterification reactions [26,41,42]. This can be attributed to the inactivating effect of the short-chain fatty acids on the enzyme activity by their irreversible linkage to the serine amino acid of the catalytic triad in the active center of the lipase, as was documented previously [43,44].

It is important to mention that the *M. echinosphaera* lipase exhibited different fatty acid preferences during the synthetic and the hydrolytic reactions. In contrast to the preference towards long-chain fatty acids during the esterification reactions, the enzyme demonstrated the highest selectivity for the C5 to C10 acids in the *p*NPP-hydrolyzing activity assays (see Section 2.2.3). Sun and Xu [41] and Sun et al. [42] also reported different fatty acid specificity in different reaction types catalyzed by the abovementioned *Rh. chinensis* lipases.

### 2.4. Immobilization of the M. echinosphaera Lipase

Physical adsorption to Accurel MP1000 was performed at 4 °C through 24-h stirring, in which the enzyme remained stable since there was no loss of activity compared to the data measured in the support-free enzyme solution. The immobilization efficiency was 91% indicating a high adsorption affinity of the lipase to the hydrophobic matrix used. The immobilized enzyme showed an activity yield of 29%, and 0.2 and 0.07 U/mg of support specific activities for *p*NPP hydrolysis and transesterification reactions, respectively. The moderate activity yield could be explained by the saturation of the support with protein during the incubation, and thereafter a protein adsorption in thick layers occurred at the end of the 24-h period, partly limiting the activity of the enzyme [45].

**Figure 7:**
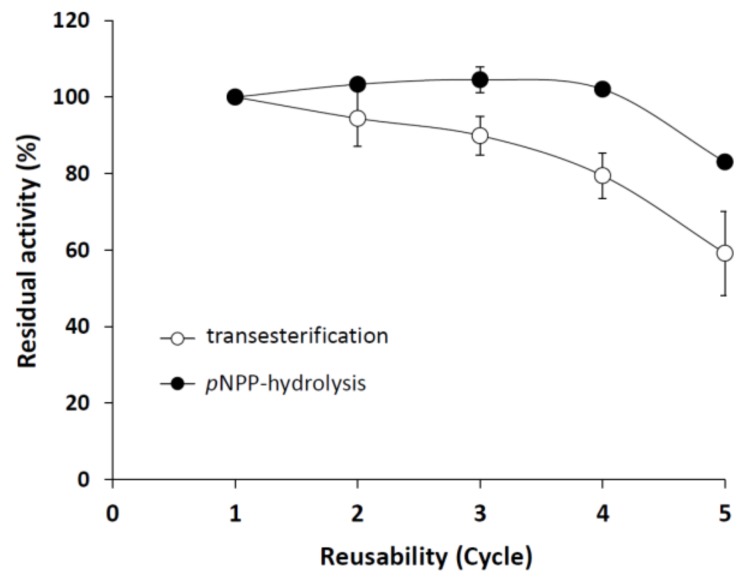
Operational stability of immobilized *M. echinosphaera* CBS 575.75 lipase during *p*NPP hydrolysis and transesterification reactions. Reaction conditions of the *p*NPP-hydrolyzing activity assay: 50 mg immobilized enzyme, 25 °C incubation temperature, 30-min incubation time, pH 6.8. Reaction conditions of the transesterification activity assay: 50 mg immobilized enzyme in 500 μL *n*-heptane, 40 °C incubation temperature, 24-h incubation under continuous shaking (200 rpm). Activity of the freshly prepared enzyme–carrier complex was taken as 100%. Values are means (*n* = 3); error bars indicate standard deviations.

The immobilized enzyme was highly stable for up to five cycles, retaining 83% and 59% of its *p*NPP-hydrolyzing and transesterification activities, respectively, at the fifth recycle step (Figure 7). The relatively high residual activity in the transesterification reaction assumes a stable *M. echinosphaera* lipase–carrier complex also in non-aqueous surroundings. For comparison, the metagenomic lipase Lip C12 immobilized on Accurel MP1000 lost all of its synthetic activity after the fourth cycle in *n*-hexane [46]. In addition, the immobilized *M. echinosphaera* lipase maintained 60% of its initial hydrolytic activity after a two-month storage at 5 °C, compared to the free enzyme that almost completely lost its activity (25% residual activity). The storage stability improving effect of the immobilization has also been described for other lipases [47]. For instance, the lipases from *Mucor miehei* (Palatase 20000L) and *Rh. delemar* (Rd) adsorbed on Accurel MP1000 were highly stable up to one and five months at 5 °C, respectively [48]. Although further studies are needed to optimize the activity yield after adsorption, our present study reported the first successful immobilization of a *Mortierella* lipase, which proved to be stable and active in both an aqueous and a water-free environment.

## 3. Materials and Methods

### 3.1. Microorganism and Submerged Fermentation

Sporangiospores (10^6^ sporangiospores/mL) of *M. echinosphaera* CBS 575.75 were transferred into two Erlenmeyer flasks (1.5 L) each containing 400 mL of mineral growth liquid medium with 2% (*w*/*v*) wheat bran and 2% (*v*/*v*) olive oil as carbon sources [13]. The cultures were incubated at 25 °C for four days under continuous shaking (200 rpm).

### 3.2. Detection of the Lipase Activities

Lipolytic and transesterification activities were determined by following standard *p*NPP (Sigma-Aldrich, Munich, Germany) based methods used in our previous works [10,23]. Unless otherwise stated, incubation temperature of 25 °C was used in the *p*NPP-hydrolyzing activity tests, while it was 40 °C for the transesterification analysis because the *M. echinosphaera* crude lipase had its maximum transesterification activity in these conditions [10]. A blank sample without the enzyme was also used in all measurements. One enzymatic unit was defined as the amount of enzyme that releases one μmol of *p*-nitrophenol in one minute under the corresponding assay conditions. Enzyme activities were measured in three independent assays.

### 3.3. Purification of Extracellular Lipase

The fermentation broth (800 mL) was filtered with gauze and Whatman No. 1 filter paper to remove fungal hyphae and wheat bran, then the crude extract was centrifuged twice at 5.040× *g* for 15 min. To precipitate the protein of the supernatant, ammonium sulfate was added in two steps up to 85% saturation. Solution was kept at 5 °C for 4 h, then precipitates were collected by centrifugation at 5.040× *g* for 15 min and re-dissolved in the smallest possible volume of 100 mM phosphate buffer (pH 6.8). Precipitate of the fraction having saturation between 50 and 85% exhibited the highest lipolytic activity. This concentrated enzyme solution was loaded onto a Sephadex G-75 (exclusion range 3 to 80 kDa; 16 × 325 mm; Sigma-Aldrich, Munich, Germany) column equilibrated with 50 mM Tris-HCl buffer (pH 8.5). Elution was performed with the same buffer at a flow rate of 0.5 mL/min. Fractions containing lipolytic activity were collected, and applied to a Macro-Prep High Q (12.6 × 40 mm; Bio-Rad, Hercules, CA, USA) anion exchange column, which was equilibrated with 50 mM Tris-HCl buffer (pH 8.5). Elution was achieved with a linear gradient of 0–1 M NaCl at a flow rate of 1 mL/min. Final purification was performed on a Sephacryl S-200 HR column (exclusion range 5 to 250 kDa; 16 × 60 mm; GE Healthcare, Uppsala, Sweden) equilibrated with 50 mM phosphate buffer (pH 6.8) containing 150 mM NaCl, and eluted with the same buffer at a flow rate of 0.5 mL/min.

### 3.4. Protein Concentration Assay

The protein content in the samples was determined by using a Qubit Fluorometer (Life Technologies, Carlsbad, CA, USA) and the Quant-iT Protein Assay Kit (Life Technologies) according to the manufacturer’s instructions.

### 3.5. Gel Electrophoresis and Zymography

SDS-PAGE was performed on 4–12% NuPage Bis-Tris gel (Life Technologies) using NuPage MES Running Buffer (pH 7.3) (Life Technologies) at 200 V. Protein bands were visualized by staining the gel with 0.2% (*v*/*v*) AgNO_3_. Molecular mass of the purified enzyme was estimated using See Blue Plus2 (Life Technologies) molecular mass standard. For zymogram analysis, the enzyme was run on a 3–12% native polyacrylamide gel (native-PAGE) using Tris-glycine buffer (pH 8.3) at 150 V. After the run, the gel was washed twice for 15 min with sodium phosphate buffer (50 mM, pH 6.8) at 4 °C. The gel was subsequently covered by 200 μM 4-methylumbelliferyl nonanoate in sodium phosphate buffer (50 mM, pH 6.8) at 25 °C. Activity bands became visible within a short time after UV illumination. Another activity staining method was also employed incubating the gel in 50 mL of sodium phosphate buffer (50 mM, pH 6.8) containing 1 mM α-naphthyl acetate (Sigma-Aldrich) and 25 mg Fast Red at 25 °C for 4 h.

### 3.6. Isoelectric Focusing

Isoelectric focusing polyacrylamide gel electrophoresis (IEF-PAGE) was performed by using Novex IEF gels (Life Technologies) containing 5% polyacrylamide and 2% ampholytes with a pH range of 3.0–10.0. Running conditions were set up according to the manufacturer’s instructions. The gel was fixed in 12% (*w*/*v*) trichloroacetic acid containing 3.5% (*w*/*v*) sulfosalicylic acid for 30 min and was stained with 0.2% (*v*/*v*) AgNO_3_. The pI of the purified lipase was determined using an IEF standard marker mix (Sigma-Aldrich) containing proteins with pI values from pH 3.6 to 9.3.

### 3.7. Characterization of the Purified Lipolytic Enzyme

#### 3.7.1. Effect of Temperature and pH

The optimum temperature of the lipase activity was determined by incubating the purified enzyme in the range from 5 to 80 °C for 30 min in 50 mM phosphate buffer (pH 6.8) containing 0.75 mM *p*NPP as a substrate. The effect of pH on the enzyme hydrolytic activity was assayed at 25 °C in a pH range of 2.2–8.0, set up by 50 mM McIlvaine buffer supplemented with 0.75 mM *p*NPP. For stability studies, the purified enzyme was pre-incubated at various temperatures (5–80 °C) for 4 h or in a McIlvaine buffer solution with corresponding pH for 24 h at 4 °C, and then residual activities were estimated after incubation for 30 min at the optimal temperature using 0.75 mM *p*NPP as a substrate. The activity assayed in the optimal temperature at an optimal pH value was recorded as 100%.

#### 3.7.2. Determination of Kinetic Parameters

The purified lipase was incubated under standard lipolytic assay conditions (pH 6.8, 25 °C and 30-min incubation time) with *p*NPP or *p*NP-decanoate substrate in concentrations ranging from 0.05 to 3.2 mM. The *K*_m_ and *V*_max_ values were calculated from the Lineweaver–Burk linear regression plot. The *k*_cat_ values were calculated by dividing the *V*_max_ by the lipase concentration of the reaction mixture.

#### 3.7.3. Determination of the Substrate Specificity

The substrate specificity of the lipase was assayed by incubating the purified enzyme with 0.75 mM *p*NP derivatives having different carbon chain-length fatty acids (*p*NP-acetate, -propionate, -butyrate, -valerate, -caproate, -caprylate, -decanoate, -dodecanoate, and *p*NPP; Sigma-Aldrich) in 50 mM sodium phosphate buffer (pH 6.8) for 30 min at 25 °C. The hydrolysis of the substrates was estimated by measuring the liberated *p*-nitrophenol. The relative rate of hydrolysis was determined as percentages of the activity obtained with *p*NPP.

#### 3.7.4. Positional Specificity Assays

Regioselectivity of the purified lipase was determined by HPLC analysis of the fatty acids released after the enzymatic hydrolysis of the synthetic 1-palmitoyl-2-oleoyl-3-linoleoyl-*rac*-glycerol (≥98%; Sigma-Aldrich) substrate. A stock solution with a concentration of 2 mg/mL was prepared by dissolving the 1-palmitoyl-2-oleoyl-3-linoleoyl-*rac*-glycerol in isopropanol. One-hundred μL of this stock, 700 μL of 50 mM sodium phosphate buffer (pH 6.8) and 200 μL of the purified enzyme solution (10 U/mL) was mixed and incubated at 25 °C for 2 h with moderate stirring in a water bath. After incubation, the mixture was extracted with 1.5 mL of hexane (VWR International, Radnor, PA, USA) for 30 s and centrifuged at 7000× *g* for 3 min. Then, the hexane layer was transferred to a HPLC vial and evaporated to dryness under a stream of nitrogen. The extraction step was repeated, and the hexane layer was collected in the HPLC vial containing the sample from the first extraction. After repeated evaporation, the extracted fatty acids were converted to fatty acid phenacyl esters. The derivatization procedure was initiated by adding 50 μL of 20 μL/mL triethylamine (Sigma-Aldrich) and 100 μL of 10 mg/mL 2,4′-dibromoacetophenone (Sigma-Aldrich) solutions diluted in acetone to the fatty acid extract. After incubation for 15 min at 85 °C, 450 μL acetone was added to the reaction mixture. The control sample contained only 1-palmitoyl-2-oleoyl-3-linoleoyl-*rac*-glycerol and subjected to the same extraction procedure described above. Phenacyl ester derivatives of fatty acids were separated on a Chromsep Polaris C18-A HPLC column (250 × 4.6 mm, 5 μm; Varian, Palo Alto, CA, USA) using the mixture of solvent A (water) and solvent B (methanol) as mobile phase at a flow rate of 0.8 mL/min. The gradient elution was performed as follows: 0.0 min, 80% B; 1 min, 80% B; 28 min, 95% B; 38 min, 100% B; 43 min, 100% B; 44 min, 80% B and 50.0 min, 80% B for re-equilibration of the column. The oven temperature and sample injection volume were 30 °C and 15 μL, respectively. The phenacyl esters were monitored at 256 nm with a Dionex UltiMate 3000 RS variable wavelength detector (Thermo Fisher Scientific, Waltham, MA, USA). Identification was performed by external standard calibration using the mixture of linoleic acid, palmitic acid and oleic acid standards (Sigma-Aldrich).

#### 3.7.5. Effect of Metal Ions and Reagents

The effect of metal ions and reagents on the lipolytic activity was examined by incubating the purified lipase in the presence of 5 mM HgCl_2_, CuSO_4_, ZnSO_4_, MnCl_2_, CaCl_2_, MgSO_4_, NaCl, KCl or CoCl_2_, or 10 mM NBS, EDTA or SDS. The residual activities were measured under standard *p*NPP hydrolysis assay (pH 6.8, 25 °C, 0.75 mM *p*NPP and 30-min incubation time), and the activity obtained in the absence of metal ions or reagents was considered as 100%.

#### 3.7.6. Effect of Organic Solvents on the Purified Lipase

The effect of various alcohols, i.e., methanol, ethanol, propanol, isopropanol, butanol, isopentanol and hexanol, and alkanes, i.e., *n*-hexane, cyclohexane, *n*-heptane and isooctane with different logP_ow_ values (from −0.85 to 4.5) on the enzyme stability and *p*NPP-hydrolyzing activity was also investigated. The purified lipase was preincubated at 25 °C for 30 min in 50 mM phosphate buffer (pH 6.8) supplemented with the corresponding solvent at concentrations ranging from 5 to 20% (*v*/*v*). Then, *p*NPP was added in 0.75 mM concentration and the mixture was incubated under standard assay conditions (25 °C and 30-min incubation time). The *p*NPP-hydrolyzing capacity of a solvent-free control sample was considered to be 100%.

### 3.8. Esterification Activity Studies

An aliquot of 300 μL of purified enzyme solution was lyophilized for 12 h in 1.5-mL screw capped vials. Then, the obtained preparation was suspended in 500 μL *n*-heptane containing 1 M methanol and 10–10 mM fatty acids (from C4 to C18, i.e., butyric, hexanoic, octanoic, decanoic, dodecanoic, tetradecanoic, hexadecenoic or octadecanoic acids; Sigma-Aldrich). Reactions were incubated with shaking (120 rpm) at 40 °C for 72 h. After incubation, mixtures were centrifuged (1500× *g*, 4 °C, 15 min) then the supernatant was collected and dried under nitrogen stream. For GC analysis, samples were resuspended in 100 μL *n*-heptane containing 0.1 mg/mL methyl heptadecanoate as internal standard. Prepared samples (2 μL) containing the internal standard were injected in split mode (split ratio was 20:1) into an Agilent 6890 N gas chromatograph (Agilent Technologies, Santa Clara, CA, USA) equipped with flame ionization detector and a HP-INNOWax (60 m × 0.25 mm × 0.5 μm; Agilent Technologies, Santa Clara, CA, USA) column under constant pressure (32 psi). During the run the oven temperature was initially 230 °C for 20 min, then increased to 240 °C with 2 °C/min and held for 25 min at the same temperature. Both the injector and detector temperature were 250 °C. The methyl ester content of the injected samples was determined by internal standard calibration using methyl ester reference compounds and methyl heptadecanoate internal standard.

### 3.9. Immobilization of the Lipase

Physical adsorption of the purified enzyme was carried out on Accurel MP1000 (particle size < 1500 μm; 3M Deutschland GmbH, Neuss, Germany) polypropylene hydrophobic support. A mass of 50 mg support was gently stirred in 20 mL 50% ethanol for 30 min to remove air from the support pores, then particles were filtered and washed with distilled water. Activated support was added to 10 mL buffered (25 mM sodium phosphate buffer, pH 6.8) enzyme solution having 33.4 U *p*NPP-hydrolyzing activity, and the mixture was incubated for 24 h at 4 °C under gently shaking (150 rpm). After incubation, the enzyme–carrier complex was filtrated from the mixture and washed several times with distilled water, then, it was lyophilized and stored at −20 °C until use. The immobilization conditions (4 °C and 24-h incubation temperature) were selected according to the studies of Manoel et al. [16] and Alnoch et al. [45]. To follow the activity loss during the incubation, a support-free enzyme solution was also prepared and incubated in the abovementioned conditions. The adsorption was monitored by measuring the residual hydrolytic activity in the supernatant, and the immobilization efficiency (IE, %) and activity yield (AY, %) were calculated based on the following equations:

IE (%) = [(A − B)/A] × 100

AY (%) = (C/A) × 100

where A is the total *p*NPP-hydrolyzing activity (U) of the enzyme solution before immobilization, B is the total *p*NPP-hydrolyzing activity (U) remained in the solution after immobilization, and C is the total *p*NPP-hydrolyzing activity (U) detected on 50 mg support under standard assay conditions (pH 6.8, 25 °C and 30-min incubation time).

Transesterification activity of the immobilized lipase was determined in capped 1.5-mL HPLC vials. Twenty-five mg of the enzyme–carrier complex was added to 500 μL *n*-heptane containing 9 mmol/L *p*NPP and 1.7 mol/L anhydrous ethanol, and the reaction mixture was incubated under continuous shaking (200 rpm) for 24 h at 40 °C. Then, the activity was determined following extraction and spectrophotometric procedures described previously [13].

To study the operational stability of the immobilized lipase, the enzyme–carrier complex was used successively in five times in hydrolytic or transesterification reactions. After each cycle, the complex was filtrated and washed thoroughly with distilled water. Then, the reaction surrounding was replaced with fresh medium. The activity of the freshly prepared enzyme–carrier complex was considered to be 100%.

## 4. Conclusions

In this work, a *M. echinosphaera* CBS 575.75 lipase with both hydrolytic and synthetic activities and desirable biochemical properties, such as broad pH stability, activity at low temperatures, and non-regioselective activity, was purified. In addition, the enzyme was extraordinarily tolerant against various polar and non-polar organic solvents and metal salts and could hydrolyze and synthesize esters with C3–C16 and C12–C18 acids, respectively. The purified lipase exhibited high adsorption affinity to the hydrophobic support Accurel MP1000 resulting in a stable, reusable, and water-free condition-tolerant enzyme–carrier complex. Characterization of the immobilized lipase and analysis of the conditions improving the activity yield after adsorption are in progress. In conclusion, the findings of this study suggest that both free and immobilized *M. echinosphaera* CBS 575.75 lipase can be efficient catalysts for applied research, especially for organic synthesis processes.

## Figures and Tables

**Table 1:** Results of purification of the extracellular lipase from *M. echinosphaera* CBS 575.75.

Purification Step	Total Protein (mg)	Total Activity (μmol/min)	Specific Activity (U/mg)	Purification Fold	Recovery (%)
Culture filtrate	2485	47,570	19.14	1	100
(NH_4_)_2_SO_4_ precipitation (50–85%)	265.6	14,548.8	54.77	2.86	30.6
Sephadex G-75	12	2672	222.67	11.6	5.6
Anion exchange (Macro-Prep High Q)	1.1	291.6	264.13	13.79	0.61
Sephacryl S200HR	0.21	79.5	378.57	19.5	0.17

**Table 2:** Effect of various metal ions and reagents on extracellular lipase activity from *M. echinosphaera* CBS 575.75.

Metal Ions and Reagents	Concentration (mM)	Residual Activity (% ± SD) ^1^
HgCl_2_	5	42.3 ± 4.5
CuSO_4_	5	104.2 ± 7.4
ZnSO_4_	5	67.0 ± 4.0
MnCl_2_	5	74.0 ± 10.5
CoCl_2_	5	81.4 ± 1.0
CaCl_2_	5	123.6 ± 5.7
MgSO_4_	5	175.1 ± 8.2
NaCl	5	137.0 ± 13.9
KCl	5	130.0 ± 9.8
NBS	10	50.4 ± 5.4
EDTA	10	142.0 ± 11.8
SDS	10	10.1 ± 1.0

^1^ The reactions were carried out at 25 °C for 30 min in phosphate buffer (pH 6.8) contained 0.75 mM *p*NPP and the corresponding metal ion and reagent compounds. The activity measured in the absence of an added compound was taken as 100%. Values represent the mean of three replicates ± standard deviation (SD).
